# Advances in preventive vaccine development against HTLV-1 infection: A systematic review of the last 35 years

**DOI:** 10.3389/fimmu.2023.1073779

**Published:** 2023-02-13

**Authors:** Carolina Souza Santana, Felipe de Oliveira Andrade, Greice Carolina Santos da Silva, Jéssica Oliveira de Souza Nascimento, Raissa Frazão Campos, Marta Giovanetti, Luciane Amorim Santos, Luana Leandro Gois, Luiz Carlos Júnior Alcantara, Fernanda Khouri Barreto

**Affiliations:** ^1^ Instituto Multidisciplinar em Saúde, Universidade Federal da Bahia, Vitória da Conquista, Brazil; ^2^ Instituto Gonçalo Moniz, Fundação Oswaldo Cruz, Salvador, Brazil; ^3^ Laboratório de Mosquitos Vetores: Endossimbiontes e Interação Patógeno-Vetor, Instituto René Rachou - Fiocruz, Belo Horizonte, Minas Gerais, Brazil; ^4^ Department of Science and Technology for Humans and the Environment, University of Campus Bio-Medico di Roma, Rome, Italy; ^5^ Escola Bahiana de Medicina e Saúde Pública, Salvador, Brazil; ^6^ Departamento de Ciências da Biointeração, Instituto de Ciências da Saúde, Universidade Federal da Bahia, Salvador, Brazil

**Keywords:** HTLV-1, vaccine, prevention, immunization, public health policies

## Abstract

**Introduction:**

The Human T-lymphotropic virus type 1 (HTLV-1) was the first described human retrovirus. It is currently estimated that around 5 to 10 million people worldwide are infected with this virus. Despite its high prevalence, there is still no preventive vaccine against the HTLV-1 infection. It is known that vaccine development and large-scale immunization play an important role in global public health. To understand the advances in this field we performed a systematic review regarding the current progress in the development of a preventive vaccine against the HTLV-1 infection.

**Methods:**

This review followed the Preferred Reporting Items for Systematic Reviews and Meta-analyses (PRISMA®) guidelines and was registered at the International Prospective Register of Systematic Reviews (PROSPERO). The search for articles was performed in PubMed, Lilacs, Embase and SciELO databases. From the 2,485 articles identified, 25 were selected according to the inclusion and exclusion criteria.

**Results:**

The analysis of these articles indicated that potential vaccine designs in development are available, although there is still a paucity of studies in the human clinical trial phase.

**Discussion:**

Although HTLV-1 was discovered almost 40 years ago, it remains a great challenge and a worldwide neglected threat. The scarcity of funding contributes decisively to the inconclusiveness of the vaccine development. The data summarized here intends to highlight the necessity to improve the current knowledge of this neglected retrovirus, encouraging for more studies on vaccine development aiming the to eliminate this human threat.

**Systematic review registration:**

https://www.crd.york.ac.uk/prospero, identifier (CRD42021270412).

## 1 Introduction

The human T-lymphotropic virus type 1 (HTLV-1) was the first described human retrovirus (1). It is estimated that around 5 to 10 million people worldwide are infected with HTLV-1 (2). Currently, the southwestern part of Japan, sub-Saharan Africa, South America, the Caribbean region, Austral-Melanesia, and some areas in the Middle East are still considered endemic regions. In the African continent, countries such as Zaire and Guinea-Bissau report the highest infection rates. In South America, Brazil has a high prevalence rate, with states located in the northeast and northern part of the country such as Maranhão, Bahia, Pernambuco and Pará, accounting the majority number of cases (3, 4).

Individuals infected with HTLV-1 may develop diseases, including the HTLV-1-associated myelopathy/tropical spastic paraparesis (HAM/TSP), the adult T-cell leukemia/lymphoma (ATLL), the HTLV-1-associated infectious dermatitis (IDH), uveitis and other inflammatory manifestation, such as arthritis, keratoconjunctivitis, and bronchoalveolitis (4, 5). Although most HTLV-1 infected individuals are classified as asymptomatic carriers (AC), some may experience non-specific symptoms such as depression and other emotional factors and have a reduced quality of life due to this infection. Furthermore, some studies have shown that there is an increased risk of mortality in HTLV-1 patients with coinfections (6–8).

HTLV-1 belongs to the genus Deltaretrovirus, and its genome encodes for structural genes such as *gag*, *pol*, and *env*. The HTLV-1 genome also has an important regulation region called pX which is flanked by two long terminal repeated regions (LTR) at its 5’ and 3’ ends (9). CD4^+^ T-cells are the main HTLV-1 target, but they can also be found in other cells, such as monocytes, B-cells, CD8^+^ T-cells, macrophages, dendritic cells, and endothelial cells (10–14). During its replication cycle, the HTLV-1 genome is integrated into the host cell genome and can induce a persistent infection. This retrovirus can alter specific cell functions, promoting a dysregulation of the infected individual’s immune system and generating overactivation and inflammation, resulting in clinical manifestations (15).

Although HTLV-1 was the first retrovirus described, associated with important diseases in humans, there is still a paucity of studies about this neglected threat. While it has been included in the sexually transmitted viruses list and within the sexually transmitted infection program of the World Health Organization (WHO), it remains a neglected disease (16). Furthermore, there is no cure for this infection and the individuals who develop the HTLV-1-associated diseases only palliative care is currently available (17). For individuals affected by ATLL specifically, there are some lines of treatment available, such as chemotherapy, and antiviral therapies in addition to the monoclonal antibody-based immunotherapy (18). At the same time, there is scientific evidence to support the use of corticotherapy in HAM/TSP patients with progressive disease, but in the same way, this treatment does not lead to a cure (19).

An aggravating factor for the control of HTLV-1 infection is the long incubation period (20). HTLV-1 carriers may remain asymptomatic for years and during this time the virus transmission can occur. In this context, a prophylactic vaccine against HTLV-1 appear to be fundamental to control the spread of this virus. Studies suggested that host and virus factors could influence the early appearance of HTLV-1-associated diseases. As an example, in the case of HAM/TSP the host gene transcription factors and the chromatin remodeling could be linked to the emergence of specific symptoms. In the case of ATLL, other factors may explain the long latency period including the breastfeeding (21, 22).

It is known that the vaccine development and its widespread deployment among vulnerable populations might have significant impacts on global public health. As an example, it is possible to see the collective world efforts to develop vaccines against the Severe Acute Respiratory Syndrome Coronavirus 2 (SARS-CoV-2) during the Coronavirus Disease 2019 (COVID-19) pandemic, which was possible due to the technological and scientific advances together with the effort within several countries. Such efforts could mediate a significant reduction in infections, morbidity, and mortality. Therefore, this study aims to analyze the current advances in the development of preventive vaccines against the HTLV-1 infection.

## 2 Methods

This systematic review was conducted following the guidelines of the Preferred Reporting Items for Systematic Reviews and Meta-analyzes (PRISMA^®^). A systematic search was performed for studies addressing the development, research, and testing of prophylactic vaccines for the HTLV-1 infection. The articles were searched in PubMed, Lilacs, Embase, and SciELO in June 2022 through the search algorithm (Human T lymphotropic virus 1 OR (HTLV-1) AND (Vaccin* or Immunization)), which is composed of Descritores em Ciências da Saúde/Medical Subject Headings (DeCS/MeSH) database subjects. All titles found in databases were cross-referenced to identify possible duplicates.

The articles found in the data platforms were initially selected by reading the title and the abstract, following the inclusion criteria: (a) original articles available in Portuguese, English, or Spanish; (b) experimental studies, in animal or human models; and (c) articles describing, developing and testing vaccination strategies against the HTLV-1. Afterward, a new selection process was performed by reading the complete manuscript. The exclusion criteria applied were: (a) articles that present only theoretical models on vaccine design; and (b) articles on therapeutic vaccines. These articles were excluded because their results could confuse the understanding of the real scenario of preventive vaccines against HTLV-1 development. The search was carried out by two independently authors (C.S.S and F.O.A) and the the presence of likely differences were discussed for a consensus with all co-authors.

Data extraction was performed by collecting the following information from each included study: (a) basic information (title, authors, year, objectives), (b) study design, (c) sample type, (d) methodology used to prepare the vaccine and (e) results of vaccine testing. The data collected were tabulated using Microsoft Excel^®^. This study was registered with the International Prospective Register of Systematic Reviews (PROSPERO) under number CRD42021270412.

## 3 Results

**Figure 1:**
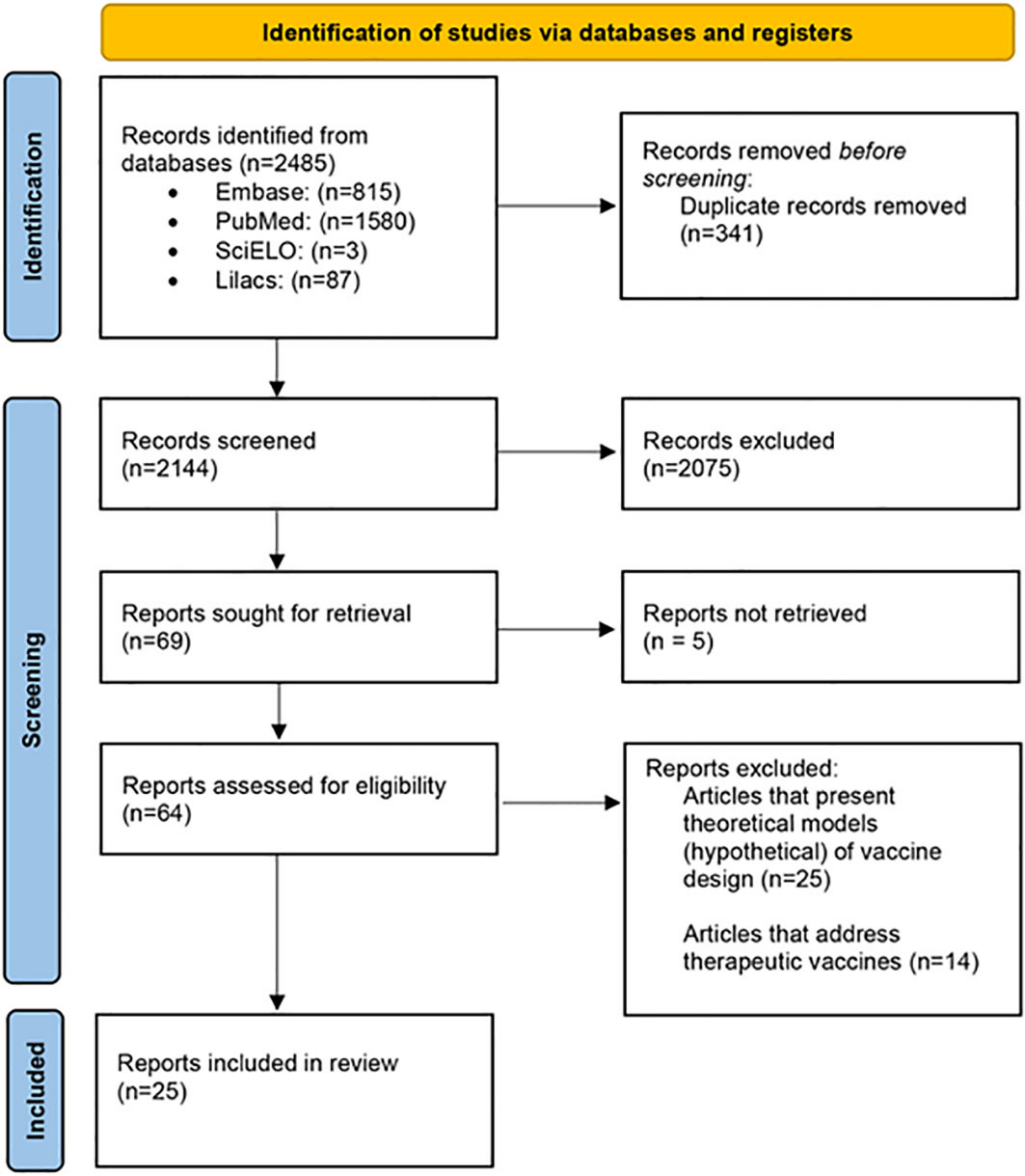
Flow diagram of the systematic selection of studies including in this review.

The systematic search found a total of 2,485 articles: 1,580 articles were available in PubMed, 815 studies in Embase, 3 articles in SciELO, and 87 in Lilacs. Among those, 341 duplicates were excluded, and 2,075 were excluded after reading both titles and abstracts. Of the 69 articles eligible for full manuscript reading, five of them were not retrieved, totalizing a total number of 64 articles eligible articles. From these, 39 articles were excluded after reading the full text and applying the exclusion criteria: (a) articles that present only theoretical models on vaccine design (n = 25); and (b) articles on therapeutic vaccines (n = 14). Thus, a total number of 25 articles were included for further screening (**Figure 1**).

Selected articles were published between 1987 and 2022. The USA was the country that submitted the majority number of articles in the field of HTLV-1 vaccine, with a total number of nine studies, followed by Japan (n=8), Iran (n=3), France (n=3), and the United Kingdom, and Canada with only one article each. All researches were experimental studies from preclinical trials. Some of the 25 articles were from the same research groups: two studies were from the Iran research group (23, 24), five articles were published by researchers from the Ohio State University (Sundaram R., et al. and Frangione et al.) (25–29) and three articles were from the same research group in France (Kazanji M et al.) (30–32).

Regarding vaccine development, some criteria are important and should be evaluated: strategic design, choice of the antigenic target, use of adjuvants, route for administration, immunization schedule, and viability (**Table 1**).

**Table 1:** Summary of the information collected on selected studies.

Reference	Authors, year	Vaccine design	Target antigen	Mode of administration	Immunization schedule	Adjuvant	Immunization time interval	Immune response assessed	Animal model	HTLV-1 challenge assessed
(33)	Ishii H., et al, 2022	Viral vector	gp46	Intranasal or intramuscular	Protocol one: 1 dose or 1 dose and 2 booster doses (0-5-6 weeks) Protocol two: 1 dose and 3 booster doses (0-4-8-9 weeks)	NI	Protocol one: 5 weeks and 1 week Protocol two: 4 weeks and 1 week	Anti-gp46 antibodies	BALB/c male mice	Challenge not rated
(23)	Kabiri M, 2018a	Peptide vaccine	p40, gp21, gp46 and p19	Subcutaneous and intranasal	1 dose and 3 booster doses (0-14-28 days)	Cpg ODN	42 days	IgG1 and IgG2a antibodies	BALB/c male mice	Challenge not rated
(24)	Kabiri M, 2018b	Peptide vaccine	p40, gp21, gp46 and p19	Subcutaneous and intranasal	1 dose and 3 booster doses (0-14-28 days)	ISCOMATRIX e MPLA	42 days	IgG1 and IgG2a antibodies	BALB/c male mice	Challenge not rated
(34)	Amirnasr M et al., 2016	Peptide vaccine	gp46	Subcutaneous and intranasal	1 dose and 3 booster doses (0-14-28 days)	Chitosan and Trimetilchitosan	NI	Anti-env13, anti-env23 antibodies	BALB/c male mice	Challenge not rated
(35)	Sugata K., et. al., 2015	Viral vector	HBZ	Subcutaneous	1 dose and 5 booster doses Mice: (0-28-49-70-91-112 days) Monkeys: (0-28-56-84-108-132 days)	Cytosine guanine phosphate	Mice:17 weeks Monkeys: every 2 weeks	CD8^+^ and CD4^+^ T-cells	NOG-SCID mice and Macaca mulatta	Challenge not rated
(36)	Mirsaliotis A., et al, 2007	Peptide vaccine	MBP-Hairpin (transmembrane glycoprotein)	Subcutaneous	3 doses (3-6-9 weeks)	Adju-Prime; Pierce	4 weeks	Monoclonal antibody	BALB/c mice and CD-1 mice	Challenge not rated
(30)	Kazanji M., et al, 2006	Chimeric peptide vaccine	gp46 and Tax	Intramuscular	Protocol one: 2 doses (0-4 weeks) Protocol two: 1 dose and 2 booster doses (6-9-16 weeks)	NI	4,6,9 e 16 weeks	Anti-env antibodies and CD8^+^ and CD4^+^ T-cells	Squirrel monkey	Inoculation with HTLV-transformed cell line, 15 days after vaccination, intravenously, negative proviral load
(27)	Sundaram R., et al, 2004a	Peptide vaccine	Tax	Subcutaneous	2 doses	N -acetil-glucosamina-3-acetil-l-alanil-d-isoglutamina (nor-MDP)	3 weeks	CD8^+^ T-cells	HHD mice	Inoculation of vaccinia virus expressing the Tax protein of HTLV-1 (p40-VV) by i.p. 10 days after vaccination with greater than 3 log reduction in viral load
(28)	Sundaram R., et al, 2004b	Peptide vaccine	gp21	NI	3 doses (3-6-9 weeks)	Muramyl dipeptide adjuvant (Nacetylglucosamine-3 yl-acetyl-L-alanyl-D-isoglutamine nor-MDP)	3 weeks	Anti-gp21 antibodies	Mices	Challenge not rated
(29)	Sundaram R., et al, 2003	Multivalent peptide vaccine	Tax	Subcutaneous	1 dose and 1 booster dose	N-acetilglucosamina-3-acetil-l- alanil d-isoglutamina (nor-MDP)	3 weeks	CD8^+^ T-cells	Mices HHD transgenics	Challenge not rated
(37)	Begum N., et al, 2002	Phage peptide vaccine	gp46	Intradermal and intramuscular	4 doses	Freund’s adjuvant	21 days	Anti-gp46 antibodies	Female rabbits	Challenge not rated
(31)	Kazanji M., et al, 2001	Peptide vaccine	gp46 and gp21	Intramuscular	Protocol one: 3 doses (0-1-3 months) and 1 booster dose (6 month) Protocol two: 1 dose	NI	6 months	Anti-env antibodies	Monkeys	Challenge not rated
(26)	Frangione-Beebe M., et al, 2001	Peptide vaccine	gp46	Intramuscular	Protocol one and two: 1 dose Protocol three: 1 dose and 1 booster dose	N-acetil-glucosamina-3il-acetil-l-alanil-d-isoglutamina (nor-MDP)	10 weeks	Anti-gp46 antibodies	New Zealand white rabbits	Challenge not rated
(25)	Frangione-Beebe M., et al, 2000	Peptide vaccine	gp46	Intramuscular	Mices: 3 doses Rabbits: 1 or 2 doses	N-acetil-glucosamina-3il-acetilL-alanil-D-isoglutamina	Mices: 3 weeks Rabbits: 10 weeks	Anti-gp46 antibodies	Mices e female rabbits	Challenge not rated
(38)	Ibuki K., et al, 1997	Peptide vaccine	gp46	Intradermal	1 dose	NI	NI	Anti-gp46 antibodies and CD8^+^ T-cells	Monkeys	Challenge not rated
(32)	Kazanji M., et al, 1997	Vector adenoviral and plasmids	gp46	Protocol one: Intramuscular Protocol two: intradermal	Protocol one and two: 3 doses (0,1,2 months) and 2 booster doses Protocol three: and 1 dose and 1 booster doses	Saponin	Group one and two: 1 month Group 3: 6 weeks	Anti-env antibodies e CD8^+^ T-cells	Female rats	Inoculation of HTLV-I-producing MT-2 cells by i.p. 12 months after immunization in protocols one and two and 13 weeks in protocol three. Only 3 to 13 individuals are not infected (PCR)
(39)	Lairmore MD, et al, 1995	Peptide vaccine	gp46	Subcutaneous	1 dose and 1 booster dose	NI	Mices: 3 weeks Rabbits: 2 weeks	Anti-gp46 and T-cells	Mices (BALB/c, C3H/ HeJ, and C57BL/6) and New Zealand white rabbits	Challenge not rated
(40)	Franchini G., et al, 1995	Viral vector (vaccinia (nyvac) and canarypox virus (Alvac)	gp46, gp63 and gp21	Intramuscular	2 doses	NI	1 month	Anti-gp46, anti-gp21 and anti-gp63 antibodies	New Zealand white rabbits	Inoculation of HTLV-1 infected cells six months after vaccination with R-ALVAC i.v., none were infected (PCR); 1 month after immunization with R-NYVAC i.m., none were infected (PCR)
(41)	Hakoda E., et al 1995	Peptide vaccine	gp46	Intradermal	1 dose	NI	NI	Anti-env antibodies	Japanese white rabbits	Blood transfusion of HTLV-I infection via i.v. 5 weeks after vaccination, only 1 of 3 individuals was not infected (PCR)
(42)	Tanaka Y., et al, 1994	Peptide vaccine	gp46	Intramuscular	4 doses	Freund's adjuvant	14 days	Anti-gp46 antibodies	C57BL6 (B6) mice, BALB/c mice, WKA rats and New Zealand white rabbits	Inoculation of HTLV-1 infected cells intravenously, none were infected (PCR)
(43)	Lairmore MD., et al, 1992	Peptide vaccine	gp46 (Env-5)	Subcutaneous	4 doses (0-2-4-6 weeks)	Freund's incomplete adjuvant or complete	2 weeks	Anti-env5 antibodies and T-cells	New Zealand white rabbits	Inoculation of HTLV-I cell lines (HTLV-I-P3 and HTLV-I-P1) by i.v. after 8 weeks of vaccination, only 1 of 7 individuals was not infected (PCR)
(44)	Ford CM., et al, 1992	Viral vector	gp46, gp21 and gp63	Intraperitoneal	NI	NI	NI	Anti-env antibodies	Balb/c, A/J and C57BU6 mice	Challenge not rated
(45)	Lal RB., et al, 1991	Peptide vaccine	gp46 (Env-5)	Intramuscular	3 doses	NI	1 month	Anti-Env-5 antibodies	Rabbit	Challenge not rated
(46)	Nakamura H., et al, 1987	Peptide vaccine	gp68 and gp46	Intradermal and intravenous	Group one: 5 doses Group two: 4 doses Group three: 3 doses	Freund’s adjuvant	Group one: 3, 1,2,3 weeks Group two: 3, 1 and 5 weeks Group three: 3,1 and 3 weeks	Anti-gp46 and anti-gp68 antibodies	Cynomolgus monkeys (Macaca fascicularis)	Inoculation of HTLV-I-producing MT-2 cells by i.v. 5 or 6 days after vaccination, group 1 and 2 did not become infected (cell culture)
(47)	Shida H., et al, 1987	Viral vector	gp46	Intradermal	NI	NI	NI	anti-gp46	Rabbits	Inoculation of HTLV-I-producing cells (MT-2) by i.v. 11 weeks post-vaccination, no individual was infected (cell culture).

### 3.1 Vaccine design

The HTLV-1 vaccine approaches described in this review can be organized into two categories: 19 studies had a peptide vaccine as a strategy, while six articles used a viral vector as a vaccine design. It is interesting to highlight that the HTLV-1 protein most used in the designed vaccine was gp46 (n=20), in addition to p40, gp21, and p19 which were evaluated in three articles. HBZ was tested in only one study. Fifteen articles analyzed in this review used adjuvants in their studies: chitosan (CHT) and n-trimethyl Chitosan (CMD); cytosine phosphate guanine (CpG); Cpg ODN (oligodeoxynucleotides containing unmethylated CpG dinucleotides); ISCOMATRIX, which is complex formulated with saponins, cholesterol, and phospholipids; and Monophosphoryl lipid A (MPLA), which is a specific agonist of TLR4. The other ten studies did not use adjuvants or did not provide information on their use.

All studies included were from animal model. Seven articles used rabbits as an animal model for testing their vaccine, four studies tested their designs with monkeys, and ten studies used mice. Three studies used both mice and rabbits to test their vaccines and one study used monkeys and mice.

### 3.2 Immunization schedule and immune response induced

Regarding the route of administration, of the 25 studies included in this systematic review, six used subcutaneous routes to administer their vaccine (27, 29, 35, 36, 39, 43). Seven studies performed intramuscular application (25, 26, 30, 31, 40, 42, 45), while three articles chose intradermal application (38, 41, 47) and one study performed the test of the vaccine in the intraperitoneal form (44). Some studies performed their tests using two vaccine delivery methodologies: intradermal and intramuscular (two studies) (32, 37), intradermal and intravenous (one study) (46), and intramuscular and intranasal (one study) (33). Finally, three studies performed their tests both subcutaneously and intranasally and evaluated their differences (23, 24, 34). However, one of the studies did not report the mode of administration of the vaccine (28).

The vaccination schedule is something that must also be evaluated during vaccine development. All the articles analyzed here developed their own vaccination protocols, but all used booster doses in their vaccination schedule. Six articles used four doses of vaccine as an immunization schedule: the first dose (day 0), followed by three booster doses (23, 24, 34, 37, 42, 43). Three studies performed three vaccine applications, with an initial dose plus two booster doses (28, 36, 45). One article evaluated a vaccine schedule with one dose and five booster doses, but with different protocols for mice and monkeys (35). Four studies applied one dose of the developed vaccine with another booster dose (27, 29, 39, 40). Two articles made their protocol with only one dose of the vaccine (38, 41). Two articles did not inform the vaccination schedule used in their research (44, 47). Seven studies used different vaccine schedules in different study groups (25, 26, 30–33, 46).

### 3.3 Protective immune responses

Of the 25 articles included in this review, 19 used a peptide vaccine and, of these, 13 analyzed the humoral immune response with the induction of antibodies against different HTLV-1 immunogens (23–26, 28, 31, 34, 36, 37, 41, 42, 45, 46); two studies looked at the cellular immune response from CD8^+^ T cells (27, 29); and four studies evaluated both the humoral and the cellular immune response (30, 32, 38, 43). Of the six articles that used viral vector vaccine, four verified the humoral immune response with induction of antibodies against different HTLV-1 immunogens (33, 40, 44, 47); one evaluated the cellular immune response from CD4^+^ and CD8^+^ T cells (35); and one verified both the humoral and the cellular immune response (32).

The humoral response was evaluated through specific antibodies: anti-gp46, anti-gp21, anti-gp63, anti-gp68, anti-env13, anti-env5, and anti-env23. Two articles only described the detection of IgG1 and IgG2 without specifically indicating the target protein (23, 24). Based on the formulation of vaccine tests in these articles, it is likely that these antibodies are specific for p40, gp21, gp46 and/or p19.

**Figure 2:**
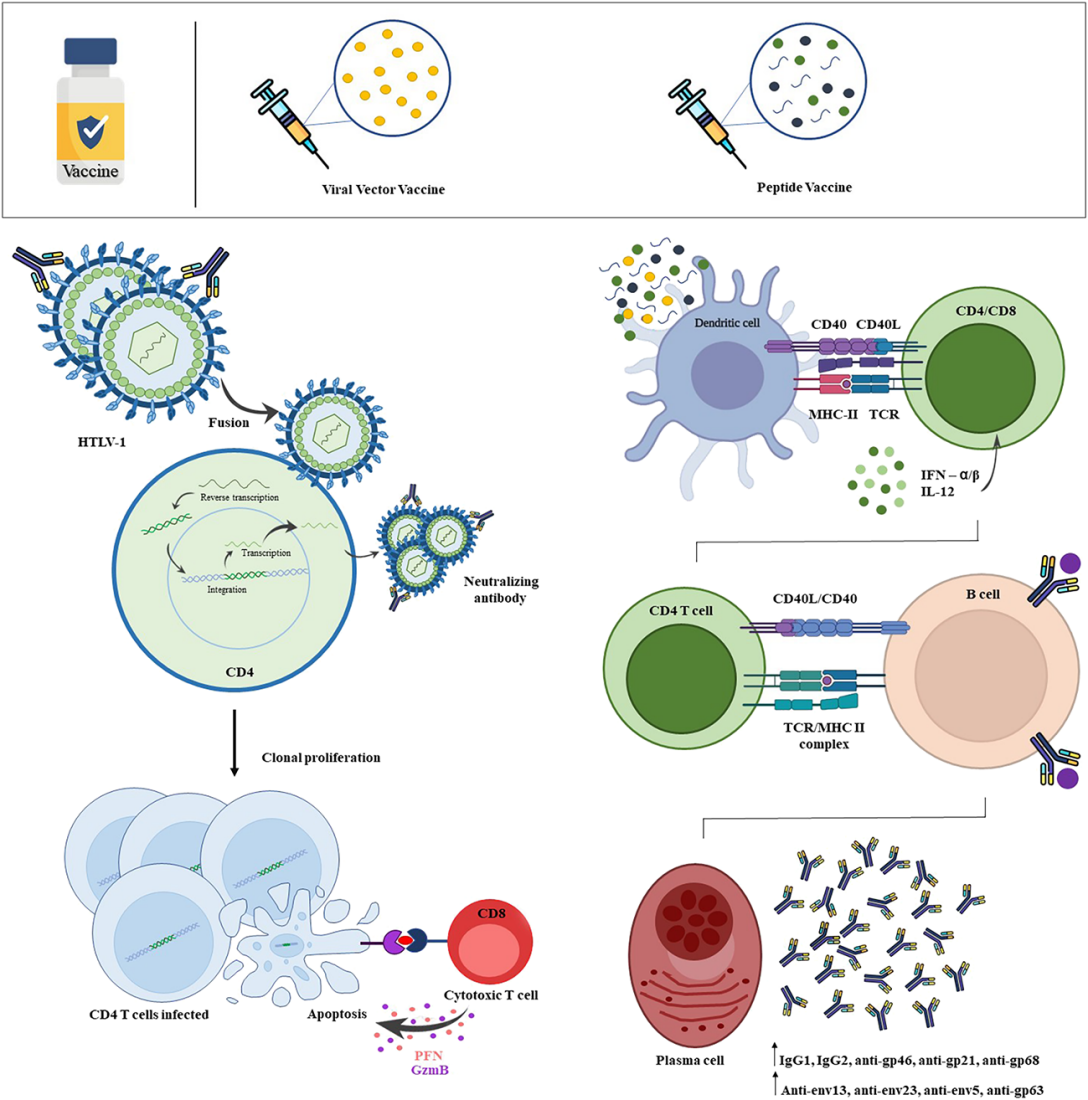
Scheme of HTLV-1 infection and vaccine-induced immune response. HTLV-1 efficiently infects T-lymphocytes, with a priority tropism for CD4+ T-lymphocytes. By infecting the host cell, viral proteins play a crucial role in the process of integrating the proviral genome into the host genome. From this, these infected cells show changes in cell behavior, inducing an intense and persistent activation, clonal expansion and cell immortalization. The vaccine-induced immune response begins from the recognition of viral antigens by dendritic cells, which will capture and present through the Class I MHC and Class II MHC complex to naives CD8+ T-lymphocytes and CD4+ T-lymphocytes, respectively. Viral antigens will also be recognized by naive B-lymphocytes, which will become activated and may present antigen to CD4+ T-lymphocyte, inducing the formation of antibody-producing plasmocytes with high affinity and memory B-cells. Thus, HTLV-1 is neutralized before binding with the host cell by vaccine-induced neutralizing antibodies, more strongly verified by the protein subunit vaccine. The viral vector vaccine was able to induce the production of cytotoxic CD8+ T-lymphocytes, that cause apoptosis of HTLV-1 infected CD4+ T-cells by release of granzyme and perforin. Created with Microsoft PowerPoint and Adobe Photoshop.

It is also important to highlight that at least nine articles found anti-gp46 induced by vaccines (25, 26, 33, 38–40, 42, 46, 47). The cellular immune response was verified in the vaccine proposals that used the TAX protein, HBZ and gp46 as an immunogen, with TAX in three studies (27, 29, 30), HBZ in one study (35), and gp46 in five articles (30, 32, 38, 39, 43) (**Figure 2**). Together those results suggest that the HTLV-1 preventive vaccine development is feasible since, after carrying out the HTLV-1 vaccination schedule, the authors verified that the hosts were protected against the infection. In addition, those studies also suggest that the gp46 can be indicated as the best immunogen to be used for future vaccine against HTLV-1, as appear to likely induce a robust cellular and humoral responses.

## 4 Discussion

Vaccination is one of the most important public health strategies for disease prevention. This systematic review highlights different HTLV-1 vaccine models capable of inducing an immune response in an animal model. The results did not demonstrate which vaccine model appear to be better to induce a high magnitude response but suggested that the development of a vaccine is feasible for preventing the HTLV-1.

The immune response against HTLV-1 is mostly mediated by CD4^+^ T-lymphocytes, cytotoxic CD8^+^ T-lymphocytes (CTL), and neutralizing antibodies. Cytokines released by CD4^+^ T helper lymphocytes (Th1) perform a fundamental role against the virus, as they augment the immune response by activating and maintaining the effector function of CTL and stimulating the neutralizing antibodies production (48). The CTL recognizes infected cells and induces apoptosis through granzyme and perforin degranulation, in addition to the FAS-FASL pathway (49). This response is important to control the proviral load. However, during the HTLV-1 natural infection, the complete mechanisms of immune response related to protection and pathogenesis is still not clear. It is known that HTLV-1 causes chronic infection in CD4^+^ T-lymphocytes, and that the CD8^+^ T-lymphocytes, mediate changes in the physiological functioning of these cells impairing the eradication of the virus (50). HTLV-1 may remain in a latent state for years in infected individuals with low replication rates and persistent inflammation, inducing several genetic changes that might trigger the HTLV-associated diseases, in particular HAM/TSP and ATLL (51). All these aspects should be considered in the design of the HTLV-1 vaccine.

The type of immune response that most effectively protects individuals infected by retroviruses is not yet clearly known. It is suggested that the immune system may respond to retroviruses from extracellular viral particles, cell-associated viruses, and endogenous genetic elements (52). Understanding how the immune response can control the HTLV-1 infection is pivotal for designing an effective vaccine. However, in retroviruses, there is an additional challenge for vaccine development due to the lack of understanding regarding both the protection and the pathogenetic mechanisms.

The highly immunogenic peptides used in vaccines are expected to stimulate the cellular immune response, creating an effector and memory-specific CD4^+^ and CD8^+^ T-lymphocytes for HTLV-1 that could control the spread of the virus from the first days of infection. In addition, it is important that vaccines can induce the production of neutralizing antibodies to mediate the virus blocking in the extracellular environment and, furthermore, induces opsonization by phagocytic cells. In this context, it is important to investigate the deleterious effects of non-neutralizing antibodies induced by vaccines, as they could bind to virus particles and facilitate viral entry into target cells, such as macrophages (53). However, the articles here analyzed did not assess the role of non-neutralizing antibodies.

### 4.1 Viral vector

One of the vaccine design methods found in this review is the viral vector vaccines. These vaccine models can be formulated to induce the expression of viral glycoproteins on the cell surface that promotes a robust specific CTL response to viral antigens (54, 55). The viral vector vaccines are based on a genetically modified virus that is deficient in its genes associated with the pathogenicity and replication cycle to optimize and personalize the desired immune responses and ensure biological safety (56). The choice of this vaccine methodology is due to some advantages, such as i) improving the expression of intracellular antigens; ii) inducing a robust response of cytotoxic T lymphocytes (CTL); and iii) stimulating the innate immune response, leading to the production of interferons and inflammatory cytokines (57, 58). However, some disadvantages must be considered, such as a pre-existing immunity to the vector and the existence of neutralizing antibodies, which can reduce the effectiveness of this type of vaccine (59).

Several human clinical trials are being conducted to produce viral vector vaccines against public health emergency infectious diseases, including the Zika virus, Influenza virus, Respiratory Syncytial Virus (RSV), HIV, malaria, and SARS-CoV-2. Some viral vectors are widely used due to their safety. These viral vectors have already been assessed and the effects on the human body are well known, such as Adenovirus and Adeno-Associated Virus Vectors (54). Nonetheless, Sugata et al. and Ford et al. chose as a viral vector the vaccinia virus (VV), considered a relatively less safe viral vector (35, 44). Another vector used was the Modified vaccinia Ankara (MVA), which can induce a strong innate immune response from Toll-like receptors (TLRs) and a robust immune response against antigens (60, 61). Although Sugata et al. used a dose of 10^7^ plaque-forming units, some studies report serious systemic adverse events at concentrations of 10^8^ (35, 62). This may be a point to consider in the development of vaccines that use MVA as a viral vector, as a disturbance in the biological safety of the vaccine could result in its non-approval for use in humans. Another article included in this review observed neutralizing antibodies from a phage clone that reacted directly with the gp46 epitope. In this sense, phages are also a more economical option for developing vaccines, since they are considered to be strongly immunogenic because they can present several T cell epitopes that are recognized in most phage strains (37). Ishii et al. used another viral vector in their vaccine project, the Sendai virus (33). This vector offers some important properties for the construction of a successful vaccine candidate, such as not being pathogenic in humans and not integrating into the host genome (63).

It is important to emphasize that for the choice of the viral vector must consider the absence of toxicity, immunogenicity, site-specific integration capability, and long-term stability (64). The first virus to be used as a viral vector was VV in the 1980s and 1990s and, at that time, it was heavily studied as a possible technique for vaccine development (65). With the studies advancement, MVA emerges as a choice in research, as the replication in mammalian cells is deficient, it is safe in laboratory manipulation and non-replicating MVA can enter any target cell. Thus, it has established itself as an extremely safe and efficient viral vector system for basic research and vaccine development (66). In this way, the studies presented in this review used vectors related to the evolution of research as time passes, since Ford et al. in 1992 used the VV while studies from the 2000s used the MVA.

Considering that previous immunity against the viral vector can reduce vaccine efficacy, the administration of booster doses can be a good strategy (54, 55). However, the study by Franchini et al. showed that even after the booster doses there was no preservation of immunization since the animals were infected with HTLV-1 after a few months (40). Moreover, the immunization schedule associated with several booster doses needs to be evaluated in the context of public health policies, as it could be a complicating factor in adherence to vaccination. The requirement of many booster doses can reduce vaccine adherence by population, as seen in the COVID-19 pandemic, whose adherence to the second and third doses of vaccines was low (67).

### 4.2 Peptide vaccine

Peptide vaccines are composed of the most immunogenic protein fragments that need to be previously selected, and are able to stimulate the humoral immune response by the production of antibodies, reducing the risk of adverse effects. The HTLV-1 envelope proteins are the primary target of the antibody response, as well as the lymphocyte CTL response (68). Neutralizing anti-Env antibodies are considered as a key to blocking the entrance of the virus in the target cells. In this review, the peptide vaccine proposals using HTLV-1 envelope protein appeared to be able to elicit the humoral immune response. Amirnasr et al. observed high titers of IgG1 and IgG2 against gp46 recombinant peptides, as well as Ibuki et al, observed a high antibody titer and the inhibition of the cell fusion activity (34, 38). Beebe et al, Lairmore et al. and Lal et al. also used the gp46 envelope protein for their vaccine design but obtained different results in their tests (26, 39, 45). Lairmore et al, despite observed a strong antibody response to the gp46, the antibodies were not able to inhibit HTLV-1-mediated cell fusion. In the article by Lal et al. it was demonstrated that antibodies induced by Env-5, can recognize the surface of infected cells, but also fail to inhibit the formation of HTLV-1 syncytia (43). Tanaka et al, Sundaram et al. and Nakamura et al. were successful in generating antibody responses against HTLV-1 with the persistence of immunization after 10 weeks, however, some schedules required booster immunizations, and the functional capacity of the antibodies was not further evaluated. Therefore, new tests with other immunodominant epitopes capable to generate a specific and robust immune response appear to be necessary.

The use of synthetic peptides that mimic important protein regions involved in the fusion between the viral cell membrane and the infected cell is an interesting strategy in the development of a vaccine directed to the HTLV-1 envelope. Sundaram et al. developed a vaccine with a coiled coil region of the HTLV-1 gp21 that induced antibodies capable of reducing cell fusion (28). However, the vaccine tested for Mirsaliotis et al, formulated with trimer-of-hairpins forming HTLV-1 transmembrane glycoproteins, was not able to block envelope-mediated membrane fusion but had complement-fixing activity (36). Previous studies have also found that antibodies directed against the coiled region of other retroviruses, such as the human immunodeficiency virus and bovine leukemia virus, can lead to neutralization (68, 69).

The vaccine models tested by Hakoda et al, Ibuki et al, Begum et al, and Sundaram et al, presented in this review demonstrated the ability to induce neutralizing anti-Env antibodies (28, 37, 38, 41)

The peptide vaccine model has an important feature. The response tends to be low and, because of this, the use of elements to enhance this response, such as the use of adjuvants, is indispensable. Moreover, synthetic peptides are also less immunogenic and require several booster doses and adjuvants (70).

Sundaram et al. present a vaccine candidate formulated from multivalent peptides with the aim of solving two problems that are associated with peptide immunogens: intracellular delivery, processing, and presentation of multiple epitopes by the same antigen-presenting cell; and the possibility of CTL epitopes degradation *in vivo*. Data suggest that this strategy appears to be interesting, as this candidate has shown the potential to overcome these limitations associated with the type of vaccine design used (29).

Kazanji M. et al. produced a chimeric peptide vaccine with Env and Tax and induced high anti-Env and anti-Tri-Tax antibody titers. This strategy may expand the immune response as it includes highly immunogenic peptides compared to the peptide vaccine used individually (30).

Frangione-Beebe M. et al, created a new technique for the encapsulation of synthetic peptide developing a gp46 subunit protein vaccine. They, with the encapsulation of Poly (d,l-lactide-co-glycolide (PLGA) microspheres, observed a longer-lasting immune response compared to a vaccine with free peptide. Thus, the use of vaccines with encapsulated peptides may be an advantageous strategy, which could need fewer booster doses (26).

Shafifar et al. present a vaccine produced with recombinant immunogenic proteins, Tax and the epitopes gp46 and gp21, for the prevention of HTLV-1, in two different ways, fusion with mouse Fcγ2a (mFcγ2a) or His-tag with adjuvants. Through the experimental challenge of this study, the authors observed that this vaccine produced distinct patterns of effector immune responses. Mice immunized with Fc-tag demonstrated the induction of Th1 cellular immune response with high production of IL-12 and IFN-γ. On the other hand, mice immunized with the His-tag immunodominant subunit presented almost the same level of protection in the challenge but demonstrated a greater production of IL-4 and, consequently, higher Th2 responses (71).

It is important to consider that protein subunit vaccines are the most commonly used approach in studies evaluating vaccine proposals against retroviruses. For example, the first clinical trial of HIV vaccines were protein subunit vaccines, as well as the first HIV vaccine tested in humans, AIDSVAX, created by Vaxgen (72, 73). However, it is questioned whether this methodology is the most suitable for retroviruses since despite inducing high production of neutralizing antibodies, it is not able to obtain a good CTL response. On the contrary, viral vector vaccines induce a good CTL response but are not able to induce a good humoral response (74). Therefore, future studies are needed to evaluate the efficacy of the immune response against different strategies for retrovirus vaccines, which ensure the induction of high antibody titers and CTL responses.

### 4.3 Theoretical models and therapeutic vaccines

In addition to the 25 vaccine candidates analyzed in this systematic review, it is important to highlight the literature studies that develop theoretical models of vaccines (75). These studies investigate the immunogenic regions of the HTLV-1 that can be used for the development of epitope-based subunit prophylactic vaccines that stimulate a robust immunological response. This, therefore, provides prospects for future effective vaccine proposals. In this way, the worldwide effort to develop vaccine platforms during the COVID-19 pandemic appears as a possibility to try to eliminate/control other viral infections, such as HTLV-1.

The improvement of tools and the application of cutting-edge bioinformatic approaches in immunology provided the bases for the development of novel vaccination strategies. Immunoinformatics, otherwise known as computational immunology, is the interface between computer science and experimental immunology. It represents the use of computational methods and resources for understanding immunological information. It combines traditional immunology with computer science, mathematics, chemistry, genomics, and proteomics for the large-scale analysis of immune system function and offers new opportunities for future bench-to-bedside research (76).

At the same time, it is known that there are currently millions of people already infected by HTLV-1 in the world. Therefore, it is important to also analyze therapeutic strategies that are able to mitigate the effects of virus infection, promoting a better quality of life for these individuals. Therapeutic vaccines may be a strategy to improve the HTLV-1-specific CTL response and to reduce the HTLV-1 proviral load promoting host-virus balance. In this way, there may be a way to prevent the emergence of HTLV-1-associated diseases or act synergistically with chemotherapy treatment and consequently improve the prognosis of serious diseases, such as ATLL and HAM/TSP.

During the selection of articles for this review, some studies have addressed therapeutic vaccines. Some of these seek to develop a safe and viable treatment option for ATLL. The promising results were: a pilot study that evaluated the response of patients to a Tax peptide (Tax-DC) pulsed dendritic cell vaccine; and a proposed vaccine expressed from the vaccinia virus, which presents an oncolytic activity that can be an effective tool in the eradication of tumors (77, 78). In this sense, Sugata K et al., 2015 also proposed a therapeutic vaccination for ATLL treatment using infected monkeys. First, they showed a prophylactic vaccination in mice with recombinant vaccinia virus (rVV) expressing mutated HTLV-1 proteins, such as TAX-M22 and HBZ-LL/AA. The immunization of mice induced specific T cell responses are able to suppress Ht48 cells (cells that express the HBZ gene at a similar level to primary ATLL cells and HBZ-Tg CD4+ T cells). Then, the authors investigated the therapeutic potential of this vaccine in monkeys (Macaca mulatta: MM557 and MM558) already infected with HTLV-1. In monkeys vaccinated with rVV, a strong CTL response and a higher frequency of IFN-γ-producing CD4^+^ and CD8^+^ T cells were observed. Therefore, the cellular immune responses to Tax and HBZ can be potentiated by therapeutic vaccination, indicating a possible way of increasing the immune response in infected HTLV-1 individuals and controlling the virus (35).

Based on the data of this systematic review, the most common target as an immunogen appear to be the gp46 and the gp21. The HTLV-1 Env proteins are genetically highly conserved among different HTLV-1 isolates and, in addition, the gp46 has functions associated with the induction of syncytia, cell-cell transmission, and antibody production, while the gp21 participates in the virus cell entry (79, 80). Furthermore, gp46 and gp21 have amino acid sequences that form linear epitopes and facilitate the binding with the monoclonal antibodies (81, 82). Therefore, gp46 and gp21 immunogens appeared as good candidates for the composition of a prophylactic vaccine against HTLV-1.

It was also shown that testing different vaccine administration routes is also critical. Some vaccine candidates induced an effective humoral response and some other are also able to produce a cellular response, making it difficult to indicate which route of administration is the ideal one. The intranasal, intradermal, intramuscular, and subcutaneous routes were able to induce antibodies against specific targets. There is still no study comparing the efficiency of different routes of administration in protecting against HTLV-1. In addition, no articles assessed the neutralizing activity of the antibodies found after vaccination. Some studies only evaluated the ability of antibodies to inhibit syncytium formation, which is not enough to determine the antibody’s ability to neutralize HTLV-1 infection. Furthermore, the evaluation of cellular immune responses induced by vaccines was not completely described by the articles included in this review. It was not possible to find details about the characteristics of the cellular immune response, such as the number of specific responder T lymphocytes, specific lymphoproliferation capacity, cytokine production, and the evaluation of cytotoxicity. For an effective prophylactic vaccine against HTLV-1 infection, it is necessary that the immunogen may be able to induce the humoral and the cellular response, in addition to a good choice of immunogenic molecules. Additionally, it is also important to challenge the immunized animals to verify the capacity of preventing HTLV-1 infection.

Although HTLV-1 was the first human retrovirus associated with the development of diseases, it is still a neglected threat, and there are many gaps in the virus knowledge that still need to be filled. This systematic review has shown a low number of studies developing and testing vaccines against HTLV-1 infection and despite the efforts, few concrete results on promising vaccines have been published. The articles bring strong and important evidence about the prophylactic proposals, despite of protective efficacy and the immune response has not been explored in deep to determine the best vaccine model against HTLV-1. At the same time, some studies show that therapeutic vaccines could be another important direction. It is also important to consider novel approaches to vaccine designs by applying modern technologies that recently showed promising, such as the mRNA vaccines. There is a need for studies that test potential vaccine strategies to protect the individual from HTLV-1 infection and treat it in cases of clinical manifestation. The gaps that still exist in this field may be associated with the discouragement of research in this area and hopefully, this review can help studies that seek to develop effective strategies capable of preventing and controlling HTLV-1 infection.

## Data availability statement

The original contributions presented in the study are included in the article/supplementary material. Further inquiries can be directed to the corresponding authors.

## Author contributions

CS: methodology, formal analysis, research, writing. FA: methodology, formal analysis, research, writing. GS: methodology, research and writing. JN: methodology, research and writing. RC: methodology, research and writing. MG: conceptualization and writing - revision and editing. LS: conceptualization and writing - revision and editing. LG: conceptualization and writing - revision and editing. LA: conceptualization and writing - revision and editing. FB: conceptualization, formal analysis, writing - revision and editing and supervision. All authors have read and approved the final manuscript.
